# Assembling Ancestors: the manipulation of Neolithic and Gallo-Roman skeletal remains from Pommerœul, Belgium

**DOI:** 10.15184/aqy.2024.158

**Published:** 2024-10-23

**Authors:** Barbara Veselka, David Reich, Giacomo Capuzzo, Iñigo Olalde, Kimberly Callan, Fatma Zalzala, Eveline Altena, Quentin Goffette, Harald Ringbauer, Henk van der Velde, Caroline Polet, Michel Toussaint, Christophe Snoeck, Laureline Cattelain

**Affiliations:** 1 Multidisciplinary Archaeology Research Institute, Department of Art Sciences and Archaeology, Vrije Universiteit Brussel, Belgium; 2 Analytical Environmental & Geo-Chemistry, Department of Chemistry, Vrije Universiteit Brussel, Belgium; 3 Department of Genetics, Harvard Medical School, Boston, MA, USA; 4 Howard Hughes Medical Institute, Boston, MA, USA; 5 Broad Institute of MIT and Harvard, Cambridge, MA, USA; 6 Department of Human Evolutionary Biology, Harvard University, Cambridge, MA, USA; 7 Department of Humanities, University of Trento, Italy; 8 BIOMICs Research Group, University of the Basque Country UPV/EHU, Vitoria-Gasteiz, Spain; 9 Department of Human Genetics, Leiden University Medical Center, the Netherlands; 10 Royal Belgian Institute of Natural Sciences, Quaternary Environments & Humans, Belgium; 11 Department of Archaeogenetics, Max Planck Institute for Evolutionary Anthropology, Leipzig; 12 ADC Archeoprojecten, the Netherlands; 13 UMR 8164-HALMA (Université de Lille, CNRS, MC) France & Cedarc/Musée du Malgré-Tout, Belgium

## Abstract

The ancient cemetery of Pommerœul, Belgium, was classified as Gallo-Roman in the 1970s’, yielding 76 cremation graves and one inhumation. However, subsequent radiocarbon analyses dated the inhumation to the Late Neolithic (4^th^-3^rd^ millennium calBC). We report osteoarchaeological analysis indicating that the inhumation was composed of bones from multiple individuals, afterwards buried as “one”. Ancient DNA analyses also finds evidence of multiple individuals and revealed another surprise: the cranium is post-Neolithic and genetically related to a pair of siblings from another Belgian Gallo-Roman site. This composite burial may have been created in Late Neolithic times, with Gallo-Romans adding the cranium, or alternatively the burial may have been fully assembled in the Gallo-Roman periods. This exceptional burial documents unexpected burial practices for both prehistoric and Roman times.

## Introduction

Post-mortem manipulations of bodies and body parts have been reported on numerous occasions in Europe in the Palaeolithic, Mesolithic and Neolithic as well as Bronze Age, Iron Age and the Roman period (e.g. Triantaphyllou 2016; Rebay-Salisbury et al. 2010; Holst et al. 2018). Such manipulations involved secondary burials, rearrangement of bodies or skeletons to make room for new bodies, selection of bones, and other practices. In Belgium, evidence of manipulation of bones is already documented in the Middle Neolithic, consisting mainly of removals, relocations, alignments, and regroupings (Cauwe 1997).

Two particularly striking examples of the manipulation of human remains include the assembling of body parts originating from different individuals discovered at the Bronze Age sites of Cladh Hallan in the Outer Hebrides in Scotland (Parker Pearson et al. 2005; Hanna et al. 2012) and of Cnip Headland on the Isle of Lewis in Scotland (Lelong 2018). In this study, we present another case of a burial of several individuals put together giving the impression of a single individual, discovered in the 1970s in grave 26 of Pommerœul, Belgium.

**Figure 1.**
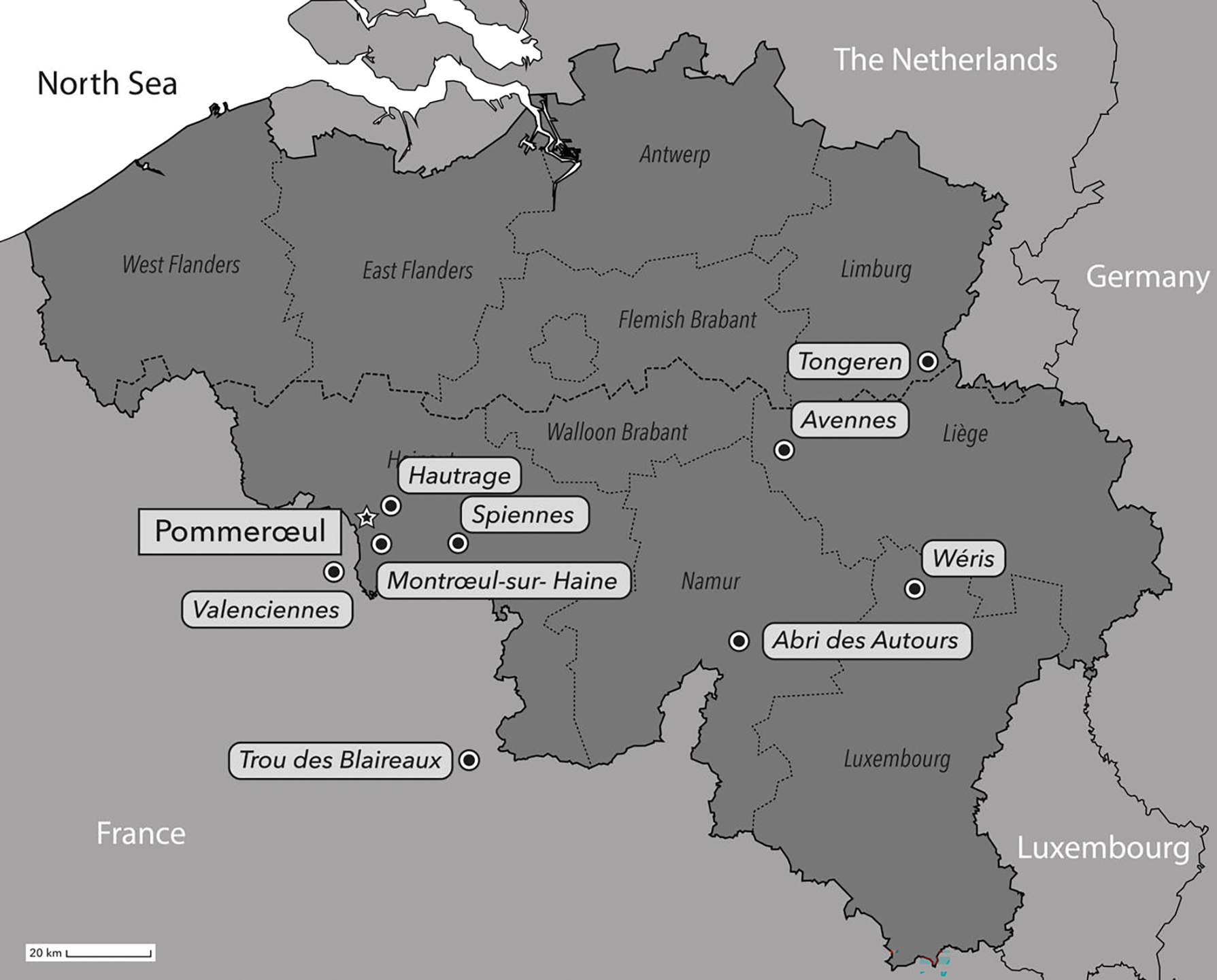
Location of Pommeroeul (star) with nearby Neolithic sites and the Gallo-Roman site of Tongeren.

Pommerœul, Belgium, is located in the western end of the Haine alluvial plain close to the French border (see Figure 1). Until recently, the earliest evidence of human settlement at this site was estimated to be mainly Late Bronze Age (1200–800 calBC; De Boe & Hubert 1977) with some traces of Neolithic occupation (Hubert 1982). Later, this area became a large and diversified Gallo-Roman harbour town comprised of working districts, a port and burial areas (Cattelain 2023).

Excavation in the 1970s of parts of the settlement and the necropolis revealed 76 cremation burials and one inhumation (grave 26). Based on the settlement characteristics, the cremation burials were dated to the Roman period (late 2^nd^ – 3^rd^ centuries AD; Cattelain 2023). The inhumation was in a deeper stratigraphic layer than the cremation deposits, and the interred individual lay in a flexed position on the right side, which is not a typical Roman burial position. Nevertheless, the preservation of the bones was excellent and the presence of a Roman bone pin near the cranium was interpreted as evidence that the inhumation was from the same period as the cremation burials. In the last few years, radiocarbon dating (Dalle et al. 2019) confirmed the cremation deposits to be Roman, but in a surprise, the dated bones from the inhumation were Late Neolithic date (see Table 1).

In the southern part of Belgium and northern part of France, the material culture of Late Neolithic *lato sensu* (3600 – 2150 calBC) is usually divided into two main phases in the sense of the French and Belgian authors (Salanova et al. 2011): Recent Neolithic (3600–3000/2900 calBC) and Late Neolithic *stricto sensu* (2900–2150 calBC). Both the Recent and the Late Neolithic are in turn subdivided whereby the second sub-phase of the Recent Neolithic (3350–3000 calBC) shows similarities to the Seine-Oise-Marne (SOM) group in the Paris Basin. The first sub-phase of the Late Neolithic is comparable to the Deûle-Escaut group in northern France and western Belgium. Later, before the end of the 3rd millennium, traces of Bell Beaker culture appear.

The Late Neolithic *lato sensu* is notable for its megalithic gallery grave (Toussaint 2003) and karst burials in limestone cliffs along the Meuse and its tributaries (Cauwe 2011). A few of the caves were also used as burial places during the Mesolithic, but the majority of these burials are more similar in their material culture to the SOM group than to the Deûle-Escaut group. The latter apparently reused megalithic tombs from the SOM, for instance in the megalithic gallery grave of Wéris; recent excavations have also revealed traces of dwellings (Hazen & Drenth 2018). Most of these caves display a combination of several funerary practices, such as primary and secondary deposits containing single and multiple individuals respectively (e.g. Abri des Autours, Trou des Blaireaux, Spiennes; Cauwe 1997) whereby the deceased individuals were often laid on the ground rather than actually being buried (Toussaint 2013). The flexed position of the Pommerœul inhumation is consistent with the style of some of these burials from Late Neolithic and also from the Early Bronze Age (Drenth et al. 2011; Veselka & Hoogland 2013; Parker Pearson et al. 2005).

Cremation was the main rite in northern Gaule in the 2^nd^ century BCE. A few burials of adults are dated to the first two centuries AD but it was not until the second half of the 3^rd^ century AD that inhumation definitively supplanted cremation which, however, was still attested at the beginning of the 4^th^ century in the settlements of Tongeren and Nervians (Hanut 2014: 82). The skeletons in Late Antiquity were generally positioned on their backs with the lower limbs straight (Blaizot et al. 2009; Mauduit *et al.* 2019) although in the first two centuries AD, more variable positions were observed with skeletons also deposited on the stomach or on the side (Blaizot et al. 2009: 37)

In this study, we deploy multiple lines of bioarcheological analysis to shed further light on the inhumation grave from Pommerœul, combining information on burial location, burial position, osteoarchaeological analysis of the skeletal elements, radiocarbon dating, carbon and nitrogen isotope, and ancient DNA analyses.

## Methods

For this study, we systematically re-evaluated the skeletal remains from grave 26. We carried out osteological sex determination using the methods outlined in the Workshop of European Anthropologists (Ferembach *et al.* 1980), evaluating the Phenice traits (Phenice 1969), and measurements of the humerus, clavicles, and femur (McCormick *et al.* 1991; Stewart 1979; Steijn & Isçan 1999). Age-at-death was estimated via changes to the pubic symphysis (Brooks & Suchey 1990) and auricular surface (Buckberry & Chamberlain 2002) and evaluating the degree of cranial suture closure (Meindl & Lovejoy 1985). The bones were macroscopically assessed for traces of human modification, using the classification as described by Bello et al. (2016; and the references therein), thereby distinguishing slicing cut marks, scrape-marks, chop marks, percussion damage, and tooth marks.

**Figure 2.**
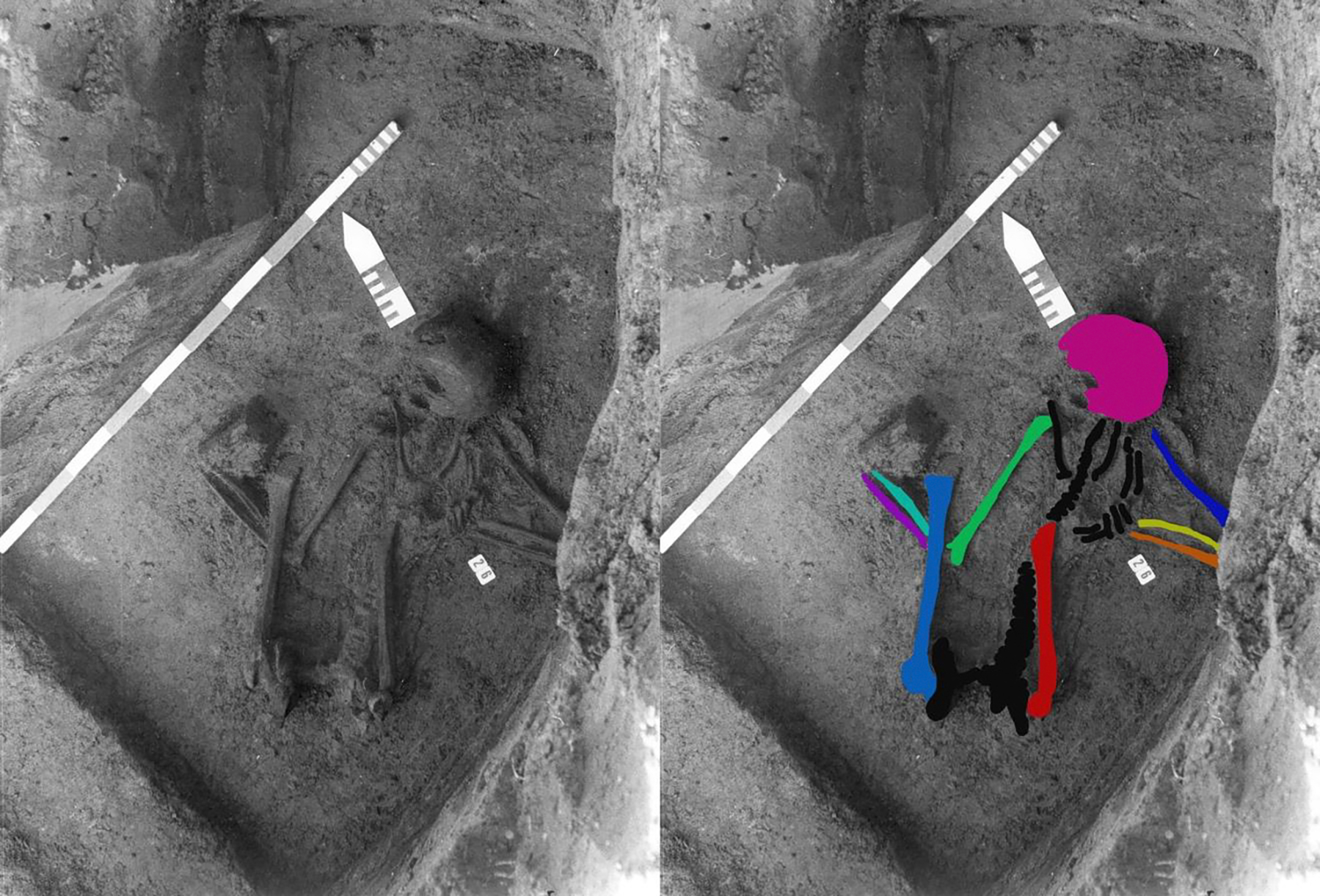
Original field photograph displaying the individual in grave 26 in anatomical articulation lying on the right side with flexed legs (left). Colour was added to the bones that were sampled for aDNA analysis (right). Tibiae, fibulae, and bones of the feet are not displayed on this photograph (with courtesy of Paumen, Wargnies, and Demory; Fédération Wallonie-Bruxelles – en dépôt Espace gallo-romain).

We sampled each of the long bones shown in Figure 2 and the five adult metatarsals for radiocarbon dating (See Appendix 1 for full details on the protocol). The same long bones sampled for radiocarbon dating as well as a second left radius, a left and right tibia, and a partial right fibula were sampled for DNA (see Appendix 1 for full details).

We generated ancient DNA data by using a sterile dentistry drill or a dental sandblasting tool to obtain approximately 37 mg of powder from each sample, working in clean room conditions at Harvard Medical School. Also in dedicated clean rooms, we extracted DNA (Rohland et al. 2018; Dabney et al. 2015; Korlević et al. 2015), and produced double- (Rohland et al. 2015) or single- stranded (Gansauge et al. 2020) libraries. We enriched the samples in-solution for both mitochondrial DNA (Fu et al. 2013) and a set of 1.24 million single nucleotide polymorphism (SNP) targets (Fu et al. 2005). We sequenced the enriched libraries on Illumina instruments, processed the sequences and aligned them to the human genome as described in previous studies (Mathieson et al. 2015), and then represented each targeted position in the genome by a single randomly chosen sequence. Results from 16 libraries we generated on 16 distinct samples are presented in Appendix 2. Of these, 8 samples (6 from Pommerœul and 2 from Tongeren) produced data on at least 3000 SNPs covering the targeted positions on chromosomes 1–22 (average about 31000, range ab out 3000–820000), and also produced contamination metrics consistent with authentic ancient DNA, and these are the new data we report in this study (Appendix 2).

## Results

The cremated remains and the inhumation from grave 26 were first analyzed in detail by Van Kerckhoven, Pigière, Ashman, and Polet in 2016 (pers. comm.). Due to the presence of a Roman bone pin near the cranium of the individual in grave 26 and the cremation deposits being Roman, the researchers assumed the inhumation to date to Roman times, which was later contradicted by radiocarbon dating. As these results were not published, the present study is the first to publish the evidence that grave 26 includes remains from multiple individuals, confirming this original observation, and also extending it.

**Figure 3.**
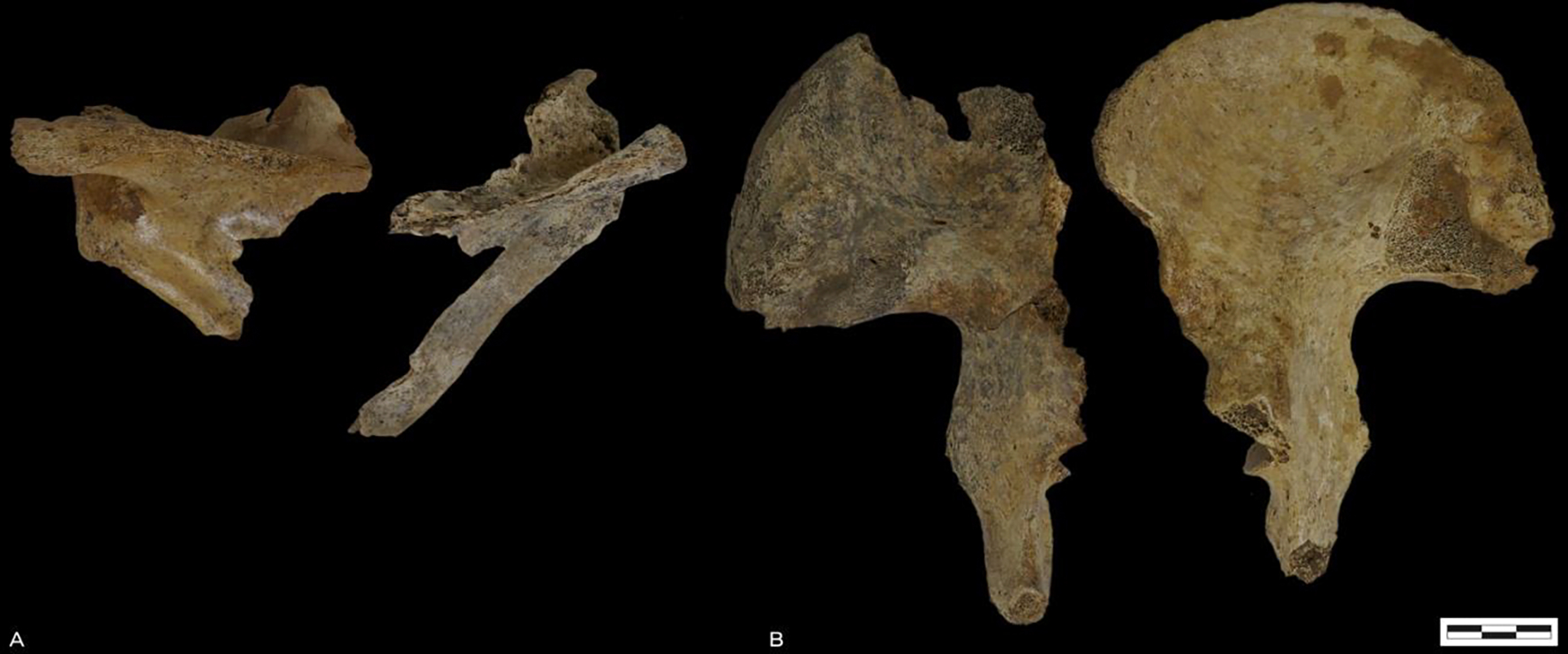
A. Left and right scapula (posterior side). B. Left and righ os coxae. Note clear shape and size differences.

In this paper, we systematically reassessed the inhumation in grave 26 and the scattered bones surrounding it. Appendix 3 presents an overview of all bones present in and around grave 26. We performed macroscopic assessment on the skeletal assemblage that was found *in situ* - tibiae, fibulae, and foot bones were not *in situ* - and show that the bones to come from multiple individuals as reflected in differences in shape and robusticity (e.g. robust vs. gracile), age (fused vs. unfused), and poor anatomical articulation (e.g. vertebrae not fitting each other). Figure 3 shows the scapulae and the pelvis whereby shape and size differences are apparent.

**Figure 4.**
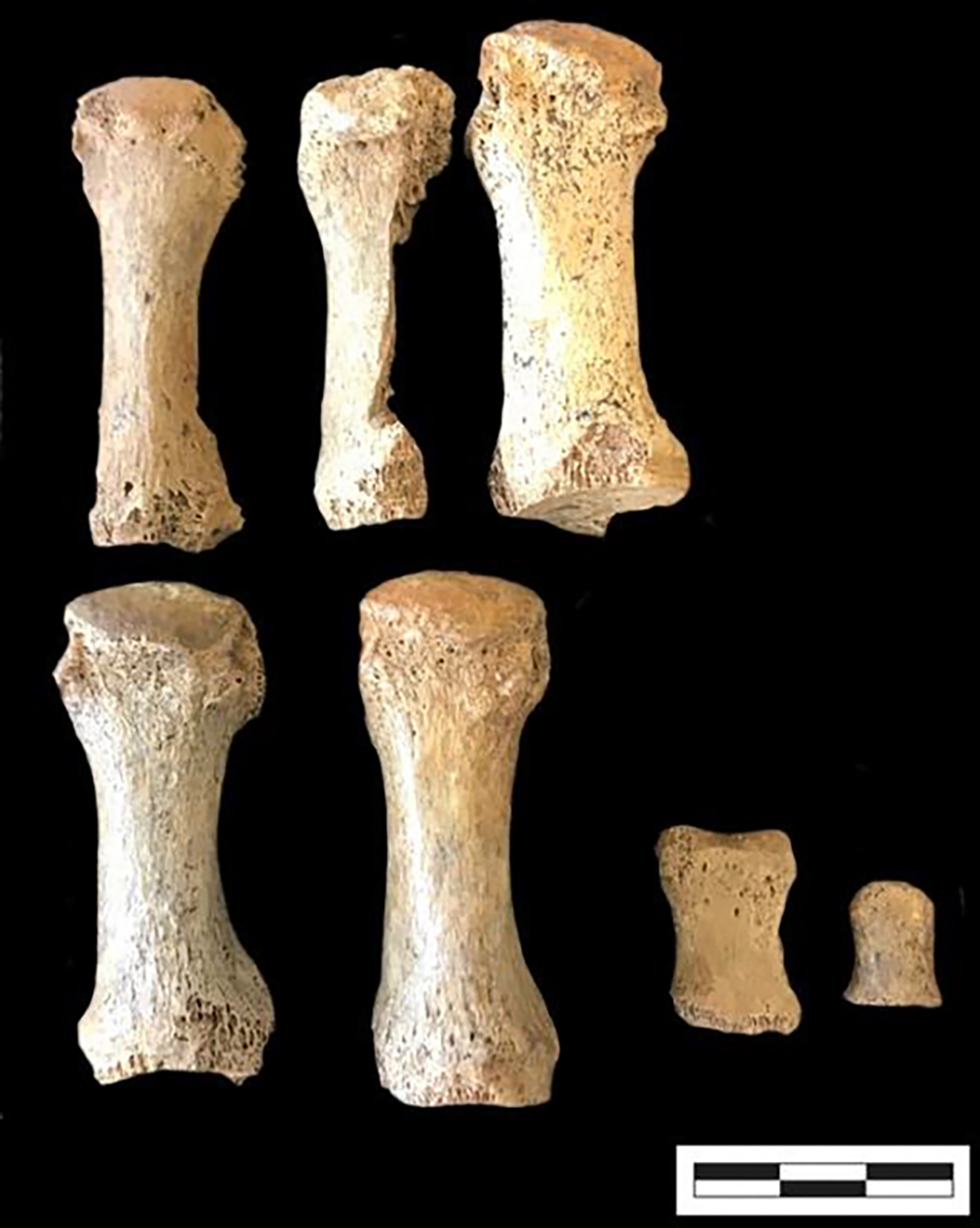
Five adult right 1^st^ metatarsals and two 1^st^ proximal foot phalanges from two different nonadults.

The osteoarchaeological analysis finds evidence of at least seven deceased Neolithic individuals, including nonadults and adults, based on the presence of five adult 1^st^ right metatarsals and the two different 1^st^ proximal nonadult foot phalanges (see Figure 4).

We were not able to determine macroscopically if all seven individual skeletons contributed body parts to the composite individual, as the metatarsals and phalanges were not in anatomical position and not visible on the original field photograph (see Figure 2). Therefore, we sampled all the long bones visible in the original field photograph as well as a second left radius, a left and right tibia, and a partial right fibula (not on the original photograph) for DNA. The DNA analysis complements the osteological one in showing that the long bones and cranium themselves come from at least 5 different individuals; it is not just the metatarsals and phalanges that are unambiguously from multiple people (Table 1).

**Figure 5.**
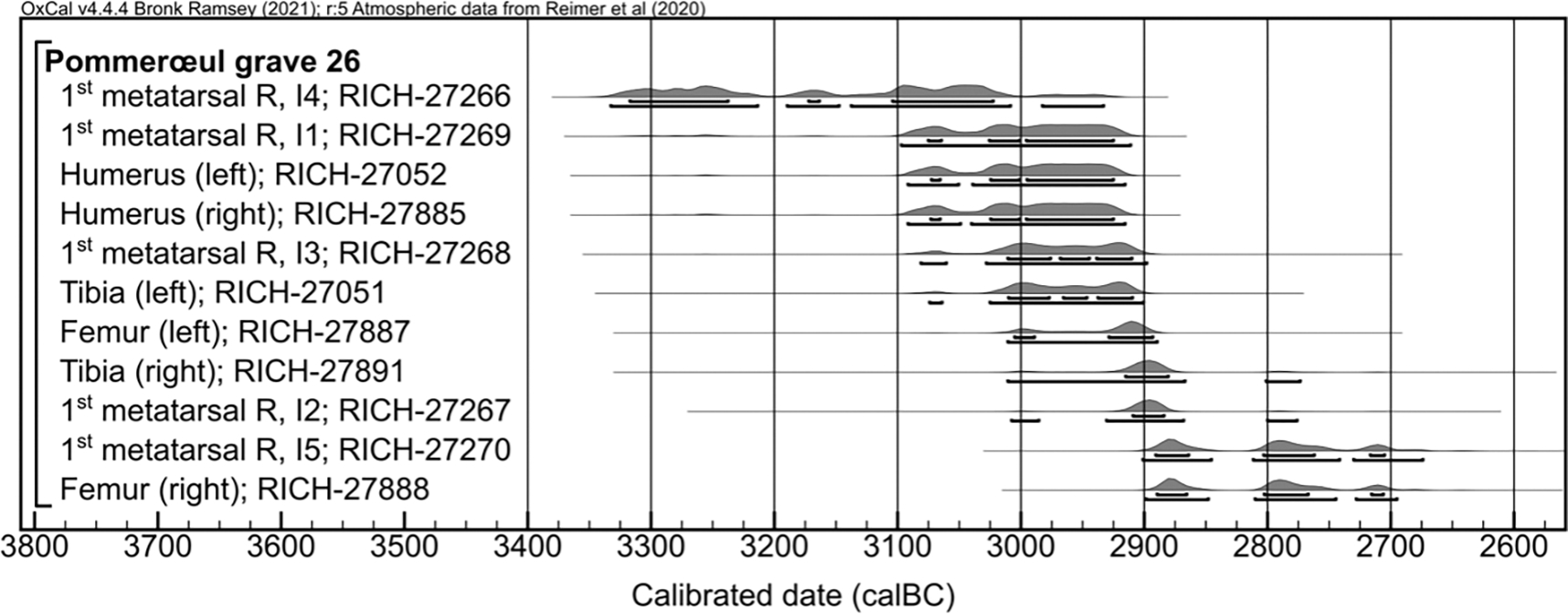
OxCal plot showing the calibrated radiocarbon dates on human bones.

We also generated additional radiocarbon dates were taken from a number of scattered bones, the badger remains, and the bone pin. The dates on some of the human bones, as presented in the calibrated dating plot, correspond to non-overlapping time intervals in the Late Neolithic (Figure 5). High variability is present especially in the dates of the metatarsals, suggesting that the individuals lived and died over at least three different times. A *χ*^2^-test reveals that the left and right femora do not seem to belong to the same individual, since they date to two non-overlapping chronological events (*χ*^2^-test: T = 8.3; p = 0.0040). The radiocarbon date from the bone pin located next to the cranium yields a Roman date, contemporary to the cremation deposits of Pommerœul. The two badger skeletal elements do not belong to the same animal, as confirmed by the differences in both the δ^**15**^N isotopes and the radiocarbon dates, with the cranium dating to the Late Mesolithic and the diaphysis to the Late Neolithic. The majority of the skeletal elements were partially covered with a black colored residue and after excavation both the human and badger skeletal remains were treated with resin impairing the assessment of whether the bones were modified. The observed indentations on a number of skeletal elements did not provide convincing evidence of human alterations of the bones. Both the resin and the black-coloured residue were removed following the protocol from Wojcieszak et al. (2020) before sample pre-treatment for ^14^C dating, as verified by FTIR analysis on the bone collagen.

Our analysis of genetic relatedness shows that the six bones successfully sampled for genetic data shows that they derived from at least five distinct individuals (Table 2). For one sample (I21565), not enough data was present to reject the possibility that it is from the same individual as the samples with the second-lowest or third-lowest amounts of data. For one of these low coverage pairs of samples, I21565-I18067, the 95% confidence interval for the relatedness coefficient is 0.49–1 which excludes zero, so these may be first degree relatives with an expected relatedness coefficient of 0.5, or from the same individual with an expected relatedness coefficient of 1. In either case, the genetic and morphological results agree in showing that the skeletal remains come from multiple individuals: at least five individuals (genetic results) and at least seven people (osteoarchaeological results).

The Principal Component Analysis (PCA) where we merged our newly reported data with previously reported ancient data (Appendix 4) and then projected onto the variation of modern individuals assessed at approximately 600,000 SNPs (Appendix 5), we found that two of the high-coverage individuals, I18068 and I21570, plot in a location midway between French Neolithic and Western Hunter-Gatherers (WHG), consistent with the expectation for an early European farmer population with high hunter-gatherer-related admixture, similar to the genetic profile of another sample of ancient DNA from the nearly lower Rhine region of present-day Germany from the Wartberg Late Neolithic culture dated to 3500–2800 BC (Immel et al. 2021) The three lower coverage individuals I21565, I18067, and I21568 are consistent with having a similar genetic profile, with their scatter around the centroid likely reflecting their limited data.

Individual I18605 with the highest coverage data plots in a very different location in PCA, close to people who lived in the Low Countries from the Late Neolithic (e.g. Netherlands Bell Beaker) to the present, with a position that shows it harbors large proportions of Steppe pastoralist ancestry that is known to have been absent in central and western Europe prior to around 2500 BCE (Olalde et al. 2018). We attempted to produce a radiocarbon date from the rest of the petrous part that yielded genetic data for this sample, but failed three times due to poor collagen preservation.

**Figure 6.**
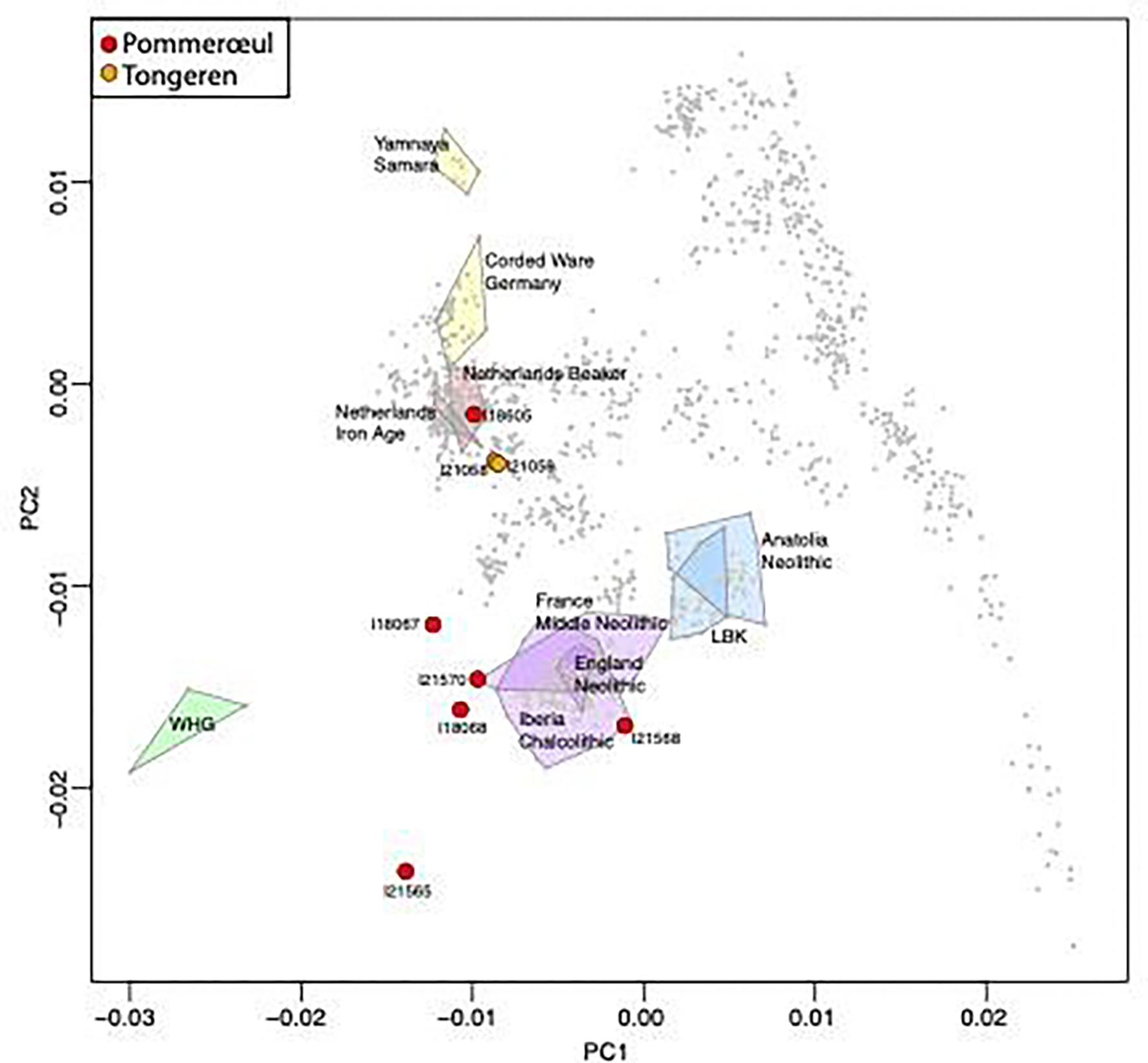
Principal Component Analysis. Projection of the genetic data from six Pommerœul samples and two Tongeren samples whose data are newly reported in this study onto genetic variation from 999 modern West Eurasians. For comparison, projected data is shown from relevant ancient groups bounded by polygons: abbreviations correspond to Western Hunter-Gatherers (WHG) and Linearbandkeramik (LBK).

Nevertheless, we obtained a date for the cranium through genetic analysis. Specifically, we used the ancIBD software to screen data from more than 10,000 ancient West Eurasian individuals with high quality genome-scale data (over 600,000 autosomal SNPs covered by at least one sequence) using exactly the dataset described in the paper publishing this method (Ringbauer et al. 2023). ancIBD searches for large segments of the genome that are genetically indistinguishable between genomes carried by both individuals (Identical-By-Descent-IBD) due to being related within a few dozen degrees of genetic separation. We identified two “genetic cousins” were identified by ancIBD, as attested by sharing large IBD segments with I18605: individuals I21509 and I21058, both from the southwestern cemeteries of the Roman (2^nd^-3^rd^ centuries AD) site of Tongeren (Belgium; > 140 km away from Pommeroeul see Figure 1) and whose data is published for the first time in this study. I18605 shares an estimated 16 centimorgan (cM) of their genome IBD with I21059, and 15 cM with I21058 (see Figure 6). These two individuals are genetic siblings as assessed by the relatedness detection methodology and indeed are consistent with sharing the same stretch of their genomes IBD with I18605 indicating that they likely inherited this segment from the same parent. Individual I21058 is a girl aged 4.5–5.5 years and I21059 is a boy aged 2–4 years and they were buried together with an adult male aged approximately 45 years, but based on uniparental marker genetic analyses the male was not the father of the pair of siblings (Van der Velde et al. 2022). Strontium and oxygen isotope analyses of the girl and the boy showed them both to have originated from the same region in which they were buried (Van der Velde et al. 2022). The girl’s radiocarbon date is 211–335 cal AD (1796 ± 24 BP; GRM15605), matching the period of the rest of the Pommerœul cremation cemetery and the Tongeren southwestern cemetery.

**Figure 7.**
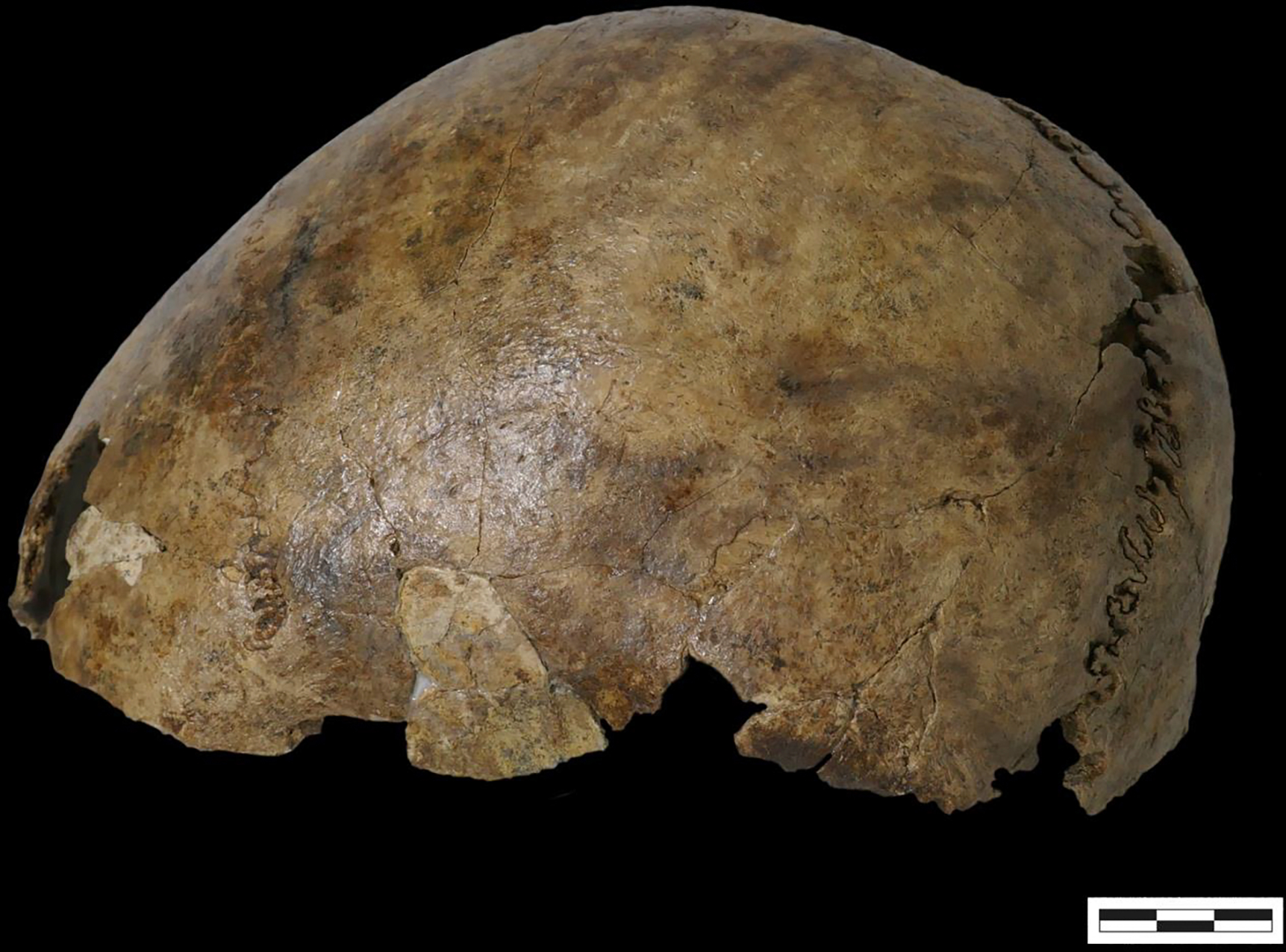
Depiction of the cranium.

The scale of IBD sharing between the two Tongeren Gallo-Roman siblings and individual I18605 is approximately what would be expected from 0–28 generations of time separation between the times they lived, or 0–784 years (the ranges correspond to 95% confidence intervals computed as described in the Methods, and the translation to years is based on an assumption of 28 years per generation (Fenner 2005; Ringbauer et al. 2023). Given that the Tongeren individuals are from the Gallo-Roman period, this corresponds to 600 BC – 1000 AD, definitely as least two and a half millennia post-dating the other Neolithic skeletal elements. Since the bone pin found at the back of the cranium dates from 68–210 AD and is consistent with the dates of the Gallo-Roman cremation graves at Pommerœul cemetery, a parsimonious scenario is that the cranium and pin were both from Roman times and were somehow combined with the much older elements in the burial feature. Although it is tempting to suggest that the anomalous genetic profile and date of I18605 reflects a sample mix-up, this is unlikely in light of the fact that among the >10,000 samples screened for IBD <1% of which were from Belgium, the only large segments of shared IBD hits were with people in another Roman site in Belgium. Although accidental mix-up could also have happened during or after storage, no other skeletal remains were retrieved from Pommerœul and the boxes are clearly marked, making such a mix-up unlikely. Figure 7 depicts the cranium (without the left os temporalis that was used for DNA and radiocarbon analyses) and when compared to the original field photograph (see Figure 3), they appear to be the same, suggesting that there was no mix-up during or after storage.

## Discussion

In Late Neolithic Europe (Cauwe 2011; Watermann & Thomas 2011), the bones of individual skeletons are usually not found in anatomical position and finding a Neolithic individual grave with the remains *in situ* is uncommon in Belgium (Cauwe 2011). Pommerœul grave 26 is remarkable considering that the interred ‘individual’ was clearly constructed from body parts of multiple individuals, as is also the case in Cladh Hallan (Parker Pearson et al. 2005; Hanna et al. 2012) and Cnip Headland (Lelong 2018).

Reports of a “single” burial being comprised of multiple individuals are very rare in general. The Middle Bronze Age site of Cladh Hallan, Scotland (Parker Pearson et al. 2005; Hanna et al. 2012) and the Early Bronze Age site of Cnip Headland (Lelong 2018) are the only examples known in Europe. In Cladh Hallan, one burial feature there, identified as male 2638, was constructed with body parts from at least three males based on bioarchaeological analyses (Parker Pearson et al. 2005), and subsequent DNA analyses of female 2613 confirmed the presence of at least three individuals (Hanna et al. 2012). Both individuals were buried in a way that suggested that they derived from a single individual (Parker Pearson et al. 2005). A composite origin also characterizes grave 26, with poorly fitting bones from several individuals were put together giving the impression of a single individual. In Cnip Headland, a selection and combination of body parts from two adults (probably female), a child, and an infant were discovered in area C. Several bones were in a somewhat anatomical position (Lelong 2018). An example from the Roman period is found outside Europe, whereby at least one composite mummy was found in the Roman cemetery of Ismant el-Kharab in Egypt consisting of body parts from two females and two children to construct a ‘single’ individual (Aufderheide et al. 1999).

### Who was responsible for assembling the new ‘individual’?

The excavators found the ‘individual’ *in situ* and the field photograph shows the ‘individual’ to have been excavated in the position that is depicted. While more extensive documentation would have been extremely valuable, the excavation occurred half a century ago and so did not employ current methodological standards. While we will never know key details of the excavation context which if they were available might highlight additional scenarios that could explain this mystery, we are grateful for the personal communication with the excavators M. Paumen, J. Wargnies, and A. Demory, and highlight below two possibilities for how this assemblage could have arisen.

One possibility raised by our findings and the archaeological context is that the Romans found this composite inhumation when burying their cremated deceased and added a cranium of one of their own accompanied by a bone pin. Either there was no cranium or the Romans completed the ‘individual’ or they replaced the existing Neolithic cranium, and in either case added the pin as a grave good. There are documented cases of the Romans disturbing tombs from other periods (Grange et al. 2020). Unfortunately due to the grave being excavated before the advent of modern archaeological methods, we do not know whether there was any evidence for recutting of the grave, which if it were documented, would provide compelling evidence in support of the scenario Roma-era modification of a Neolithic grave.

As second possibility is that the Romans assembled the ‘individual’ entirely, combining Neolithic skeletal remains they found nearby with a cranium from their own period. To our knowledge, this would be the first Roman grave were a new ‘individual’ was assembled and in which both prehistoric and Roman bones were used.

To what extent are each of these scenarios plausible? A flexed position is rare but not unusual in the middle and final Neolithic, for example, it is documented at Avennes a few tens of kilometers east of Pommerœul (Destexhe-Jamotte 1947, 1959; Figure 1). However, it is unknown in the regional Gallo-Roman period (Blaizot et al. 2009; Mauduit *et al.* 2019). These considerations add weight to the scenario that the composite burial was first assembled by a local Neolithic group and that some 2500 years later, the Gallo-Roman population restored the composite burial that their cremation burials disrupted. However, the scenario of a Gallo-Roman assemblage with scattered Neolithic bones cannot be ruled out considering the documented cases of handling human remains and the reverence the Roman-era people had for the deceased (Grange et al. 2020).

The badger remains were perhaps deposited as meaningful grave goods, although only the immature bone is contemporary with part of the human bones deposited (Table 1). The adult bone is much older, which would suggest the reuse and deposition of an old badger bone as part of the burial. The badger is a burrowing species and it is possible that these elements represent parts of carcasses of animals that died of natural causes at the location, although another possibility is that the digging of the grave pit disturbed an existing badger burrow. Even more enigmatic is the presence of the burnt badger phalanx, which is not consistent with natural arrival and documents that the presence of badger remains is at least in part due to human activity.

### Where are the remaining parts of the skeletons?

Beyond the dated bones as Tongeren, the nearest sites where Neolithic human bones have been found are the Neolithic flint mines from Spiennes, Belgium (Toussaint et al. 2019), and Valenciennes, France (Deckers & Delassus 2009), both some 20 km from Pommerœul (Figure 1). If the remains were transported from places like these, this would likely have happened well after the time of their primary burial, as no cut or chop marks were observed on the bones (Robb et al. 2015; Bello et al. 2016), indicating either that they were already defleshed and cutting them loose from the body was not necessary or the bodies would have been decomposed enough that cutting body parts loose would not necessarily have left a mark (e.g. Domínguez-Rodrigo 2003).

It is also possible that the Neolithic skeletons were local burials at Pommerœul, suggesting that the rest of the skeletons still may be in the vicinity. The pit that allowed the excavation of grave 26 is relatively narrow and the remainder of the skeletons could even still be present in the surrounding area that was not further excavated. A few flint finds in the cemetery of Pommerœul and the surrounding areas of Montrœul-sur-Haine and Hautrage are consistent with the presence of people already in Neolithic times (Dufrasnes 1999; 2001; Dufrasnes et al. 2021), even though at Pommerœul itself settlement layers are only documented from the Late Bronze Age. Unfortunately, no teeth were preserved allowing the analysis of strontium and oxygen isotopes, which would provide insight in mobility patterns.

The remaining bones from the genetic female who was the origin of the cranium are also a mystery. Although a combination of cremation and inhumation was common in the Roman period (Hollevoet 1993; Van der Velde et al. 2022), only cremation deposits were retrieved outside of grave 26. A plausible scenario is that the rest of the female’s skeleton was cremated and added as a cremation deposit among the others in the cemetery. All the cremation deposits contain cranial fragments, apart from T25, T51, T60 and T87 (Veselka et al. 2023). T25 is particularly a good candidate, as it lies immediately adjacent to grave 26. The total weight of the cremation deposit from T25 is low (23.9 g), implying that either only parts of her skeleton were burned and buried or that only some of her burned remains were buried in T25, while the rest was potentially distributed among one or more of the other cremation deposits. Analysis of the cremation deposits yielded 11 deposits with 2 or more individuals (Veselka et al. 2023) and although none of the total weights superseded 1700 g, it is indeed possible that the rest of the potentially cremated females remains were divided among the other cremation burials parallelling the composite nature of the inhumation burial. Alternatively, consistent with other funerary practices in the Roman period (Grange et al. 2020), the rest of the female’s skeleton could have been inhumed in the vicinity.

### Why was this ‘individual’ assembled?

A flexed position is documented in Late Neolithic and Bronze Age burials all over Europe (e.g. Drenth et al. 2011; Rathmann et al. 2022; Bourgeois & Kroon 2017) and the ^14^C dates of the post-cranial bones are Neolithic. If indeed a Neolithic population assembled the ‘individual’, it is notable that most of the skeletal samples used to assemble it were from individuals who were not closely related, implying that the ‘individual’ may have fulfilled a need of a group of people that were not genetic relatives but potentially considered themselves as such, as suggested for the Cladh Hallan remains (Parker Pearons et al. 2005). It is tempting to hypothesize that the “individual” was intended to posthumously represent, defend, or connect them to either other living individuals, such as neighboring families or tribes, or deceased individuals, such as ancestors as it was also postulated by Lelong (2018) for the case of Cnip Headland. A m motivation to connect to the afterlife can also be hypothesized for the Gallo-Roman population, who potentially wanted to make amends for the disturbance of the grave or constructed a new individual with agency in the afterlife. The ancient Roman attitude towards death was that the deceased never stopped being part of community life (e.g. Erasmo 2001; Parker Pearson 2008) and the handling of the human remains was part of the ritual surrounding death (Graham 2009). Whether the skeletons were initially buried at Pommerœul or stored elsewhere, and whether the burial composition occurred in the Late Neolithic or the Roman period, this group of people organized the selection of bones, decided upon a fitting location, and placed each of the bones (as displayed in Figure 2) in the correct anatomical position. Not only does this imply highly developed organizational skills, but also a certain degree of knowledge of human anatomy. The Gallo-Roman individuals of Pommerœul appear to have at least added to the composite individual. Whether they were inspired by superstition or felt the need to connect,but suggests that we should develop an even broader view of the range of both Neolithic and Gallo-Roman burial rites. These results also highlight the importance of reevaluating old collections of human remains.

## Supplementary Material

Appendix 1. Materials and Methods

Appendix 2. Online Table 1 (ancient library details) revised

Appendix 3. Online Table 2 (published modern individuals used in PCA)

Appendix 4. Online Table 3 (published ancient individuals used in PCA)

Appendix 5. Overview skeletal elements_

## Figures and Tables

**Table 1.** Overview of DNA, radiocarbon dates, and carbon and nitrogen isotope results per skeletal element.

Skeletal element	Colour in Fig. 2	Lab Code DNA	Sex	Kinship	Lab Code ^14^C	^14^C Age BP ± σ	Age cal BC/AD (2 σ)	δ^13^C	δ^15^N	C/N
PP-R	Magenta	I18605	F	n/a	n/a	n/a	n/a	n/a	n/a	n/a
H-L	Dark blue	I18067	M	No	RICH-27052	4388 ± 26	3092 – 2916 calBC	−21.1	9.5	3.2
H-R	Green	I21573	U	U	RICH-27885	4388 ± 27	3092 – 2916 calBC	−20.6	8.9	3.2
R- L (gracile)	Yellow	I21564	M?		n/a	n/a	n/a	n/a	n/a	n/a
R-L (robust)	ND	I21565	M	No	n/a	n/a	n/a	n/a	n/a	n/a
R-R	Turquoise	I21566	U	U	n/a	n/a	n/a	n/a	n/a	n/a
U-L	Orange	I21567	U	U	n/a	n/a	n/a	n/a	n/a	n/a
U-R	Purple	I21568	U	U	n/a	n/a	n/a	n/a	n/a	n/a
Fe-L	Red	I18068	M		RICH-27887	4320 ± 27	3011 – 2890 calBC	−20.9	9.5	3.2
Fe-R	Blue	I21572	U	U	RICH-27888	4212 ± 26	2899–2696 calBC	−21.2	9.4	3.3
T-L	ND	I21569	U	U	RICH-27051	4351 ± 27	3075 – 2901 calBC	−21.0	9.0	3.3
T-R	ND	I21570	F	No	RICH-27891	4278 ± 27	3017 – 2906 calBC	n/a	n/a	n/a
Fi-R	ND	I21571	U	U	n/a	n/a	n/a	n/a	n/a	n/a
1^st^ MT-R I1	ND	n/a	n/a	n/a	RICH-27269	4389 ± 31	3098 – 2912 calBC	−20.9	9.3	3.2
1^st^ MT-R, I2	ND	n/a	n/a	n/a	RICH-27267	4276 ± 31	3008 – 2777 calBC	−21.1	9.1	3.2
1^st^ MT-R, I3	ND	n/a	n/a	n/a	RICH-27268	4352 ± 32	3082 – 2899 calBC	−21.2	8.8	3.3
1^st^ MT-R, I4	ND	n/a	n/a	n/a	RICH-27266	4445 ± 31	3333 – 2934 calBC	−20.5	9.5	3.2
1^st^ MT-R, I5	ND	n/a	n/a	n/a	RICH-27270	4213 ± 31	2902 – 2675 calBC	−21.0	10.3	3.3

Animal skeletal element										
Badger cranium	ND	n/a	n/a	n/a	RICH-29393	6964 ± 31	5971 – 5746 calBC	−19.8	7.6	3.2
Badger Humerus	ND	n/a	n/a	n/a	RICH-29394	4715 ± 25	3625 – 3375 calBC	−20.2	10.1	3.3
Bone pin	ND	n/a	n/a	n/a	RICH-29395	1907 ± 22	69 – 210 AD	−22.9	8.3	3.3

PP-R = petrous part right, F = female, n/a = not applicable, H-L = humerus left, M = male, H=R = humerus right, U = unobservable, R-L = radius left, R-R = radius right, U-L = ulna left, U-R = ulna right, Fe-L = femur left, Fe-R = femur right, T-L = tibia left, T-R = tibia right, Fi-R = fibular right, 1^st^ MT-R = first metatarsal right, I = individual.

**Table 2.** Relatedness matrix.

		ID1	I21565	I21568	I18067	I18068	I21570	I18605
		ID2	T26-E	T26-H	T26-B	T26-C	T26-J	T26-A
ID1	ID2	SNPs	2981	4345	16795	84775	159871	616997

**I21565**	T26-E	2981	.	0–1	0.49–1	0–0.37	0–0.28	0–0.22
**I21568**	T26-H	4345	.	.	0–0.66	0–0.28	0–0.22	0–0.10
**I18067**	T26-B	16795	.	.	.	0–0.14	0–0.10	0–0.08
**I18068**	T26-C	84775	.	.	.	.	0–0.04	0–0.03
**I21570**	T26-J	159871	.	.	.	.	.	0–0.02
**I18605**	T26-A	616997	.	.	.	.	.	.

The number of SNPs covered at least once is shown, 95% confidence intervals of the relatedness coefficient are highlighted in orange (cases where identical genetics corresponding to a value of 1 can be excluded) or red (cases that cannot be excluded as the pair being from the same individual). Limited data means that it is not possible to determine whether the lower coverage sample of the six from which ancient DNA was obtained (I21565) is different from the second or third lowest coverage individuals (full relatedness cannot be excluded in these cases). Genetic data is present from at least five distinct individuals.

## References

[R1] Aufderheide AC, Zlonis M, Cartmell LL, Zimmerman MR, Sheldrick P, Cook M and Molto JE. 1999. Human Mummification Practices at Ismant el-Kharab. The Journal of Egyptian Archaeology 85: 197–210.

[R2] Aufderheide AC, Cartmell LL, Zlonis M, Sheldrick P. 2004. Mummification practices at Kellis site in Egypt’s Dakhleh Oasis. Journal of the Society for the Study of Egyptian Antiquities 31: 63–77.

[R3] Bello SM, Wallduck R, Dimitrijević V, Živaljević I, Stringer CB. 2016. Cannibalism versus funerary defleshing and disarticulation after a period of decay: comparisons, of bone modifications from four prehistoric sites. American Journal of Biological Anthropology 161: 722–743. 10.1002/ajpa.23079 27561127

[R4] Blaizot F, Bel V, Bonnet C, Wittmann A, Vieugué J, Deberge Y, Georges P et Gisclon J-L. 2009. La pratique de l’inhumation. In : Blaizot F (dir.), Pratiques et espaces funéraires de la Gaule durant l’Antiquité. Gallia Archéologie de la France antique, CNRS Éditions 66: 15–87.

[R5] Bourgeois Q, Kroon E. 2017. The impact of male burials on the construction of Corded Ware identity : reconstructing networks of information in the 3rd millenium BC. PLoS One 12 : e0185971 29023552 10.1371/journal.pone.0185971 PMC5638321

[R6] cattelain L 2023. La nécropole gallo-romaine sud de Pommerœul (province de Hainaut, Belgique). Viroinval: Cedarc.

[R7] Cauwe N 1997. Bibliographie raisonnée des sépultures collectives de la Préhistoire de Belgique. Bulletin de la Fédération des Archéologues de Wallonie 47: 112. Brussels: Fédération des Archéologues de Wallonie.

[R8] Cauwe N 2011. La fin du Néolithique, in Cauwe N, Hauzeur A, Jadin I, Polet C, Vanmontfort B (eds.) 5200–2000 av. J.-C. Premiers agriculteurs en Belgique: 65–70. Viroinval: Cedarc.

[R9] Dabney J, 2015. Complete mitochondrial genome sequence of a Middle Pleistocene cave bear reconstructed from ultrashort DNA fragments. PNAS 110: 15758–15763. 10.1073/pnas.1314445110 PMC3785785 24019490

[R10] Dalle S 2019. Preliminary Results in the Collecting of Protohistoric Cremation Samples for the CRUMBEL Project. Lunula : Archaeologia Protohistorica 27: 9–14.

[R11] De Boe G & Hubert F. 1977. Une installation portuaire d’époque romaine à Pommerœul. Archaeologia Belgica 192. Brussels: Service national des fouilles.

[R12] Deckers M & Delassus D. 2009. Valenciennes, vallée de l’Escaut. Un site du Néolithique final Valenciennes: Service archéologique de Valenciennes.

[R13] Destexhe-Jamotte J 1947. La sépulture néolithique d’Avennes (province de Liège). Époque Robenhausienne, Bulletin de la Société royale belge d’Anthropologie et de Préhistoire 58: 8–19.

[R14] Destexhe-Jamotte J 1959. Le Néolithique de la vallée de la Méhaigne (Hesbaye Liégeoise), Bulletin de la Société royale belge d’Anthropologie et de Préhistoire 70: 17–63.

[R15] Devièse T, Abrams G, Hajdinjakd M, Pirson S, De Groote I, Di Modica K, Toussaint M, Fischer V, Comeskeya D, Spindlera L, Meyerd M, Semal P & Highama T. 2021. Reevaluating the timing of Neanderthal disappearance in Northwest Europe. PNAS 118, n° 2. 10.1073/pnas.2022466118 PMC7999949 33798098

[R16] Drenth E, Meurkens L, Van Gijn AL. 2011. Laat-Neolithische graven 209–280. Leiden: Archol BV & ADC ArcheoProjecten.

[R17] Dufrasnes J 1999. Quelques objets, datant de la préhistoire à la période moderne, découverts dans les déblais du canal à Pommerœul. Vie Archéologique 52: 29–60.

[R18] Dufrasnes J 2001. Petit matériel, d’époque diverses, mis au jour à l’occasion du creusement du canal à Pommerœul en 1975. Vie Archéologique 55–56: 27–48.

[R19] Dufrasnes J, Leblois E, Picavet O. 2021. La villa gallo-romaine du “Ruissaeu de Villers” à Hautrage/Villerot (Belgique, Hainaut). Bulletin de la Société tournaisienne de Géologie, Préhistoire et Archéologie 17: 59–110.

[R20] Erasmo M 2001. Among the dead in ancient Rome. Mortality 6: 31–43.

[R21] Fenner JN 2005. Cross-cultural estimation of the human generation interval for use in genetics-based population divergence studies. American Journal of Biological Anthropology 128: 415–423. 10.1002/ajpa.20188 15795887

[R22] Fu Q, 2005. An Early Modern human from Romania with a recent Neanderthal ancestor. Nature 524: 216–219. 10.1038/nature14558 PMC4537386 26098372

[R23] Fu Q, 2013. DNA analysis of and Early Modern human from Tianyuan Cave, China. PNAS 110: 2223–2227 23341637 10.1073/pnas.1221359110 PMC3568306

[R24] Gansauge MT, Aximu-Petri A, Nagel S, Meyer M. 2020. Manual and automated preparation of single-stranded DNA libraries for the sequencing of DNA from ancient biological remains and other sources of highly degraded DNA. Nature Protocols 15: 2279–2300 32612278 10.1038/s41596-020-0338-0

[R25] Grange G, Charbouillot S, Silvino T. 2020. Réouvertures de tombes dans la nécropole antique de Saint-Vulbas (Ain) in Ritualiser, gérer, piller. Rencontre autour des réouvertures de tombes et de la manipulation des ossements. Actes de la 9e rencontre du Gaaf Poitiers 231–239. Poitiers: APC Mémoire LII.

[R26] Hanna J, Bouwman AS, Brown KA, Parker Pearson M, Brown TA. 2012. Ancient DNA typing shows that a Bronze Age mummy is a composite of different skeletons. Journal of Archaeological Science 38: 2774–2779.

[R27] Hanut F 2014. Le passage de la crémation à l’inhumation dans le Nord de la Gaule. In : Hanu F, Henrotay D, ”Du bûcher à la tombe”. Catalogue de l’exposition organisée au Musée archéologique d’Arlon du 24 octobre 2014 au 22 mars 2015. Namur, Institut du Patrimoine Wallon: 82–84.

[R28] Hazen PLM, Drenth E. 2018. A Late Neolithic site of the Deûle-Escaut Group (?) with two probable house-plans at Eine-Heurnestraat (mun. of Oudenaarde, East Flanders, BE). Notae Praehistoricae 38: 89–98.

[R29] Hubert F 1982. Site portuaire de Pommerœul. I. Catalogue du matériel pré - et protohistorique. Archaeologia Belgica 278.

[R30] Hollevoet Y 1993. Ver(r)assing in een verkaverling. Romeins grafveld te Oudenburg (prov. West-Vlaanderen). Archeologie in Vlaanderen 3: 207–216.

[R31] Holst MK, Heinemeier J, Hertz E. 2018. Direct evidence of a large Northern European Roman period martial event and post-battle corpse manipulation. PNAS 115: 5920–5925. 29784805 10.1073/pnas.1721372115 PMC6003345

[R32] Immel A, 2021. Genome-wide study of a Neolithic Wartberg grave community reveals distinct HLA variation and hunter-gatherer ancestry. Communications Biology 4: 113 33495542 10.1038/s42003-020-01627-4 PMC7835224

[R33] Korlević P, 2015. Reducing microbial and human contamination in DNA extractions from ancient bones and teeth. Biotechniques 59: 87–93. 26260087 10.2144/000114320

[R34] Le Cabec A, Toussaint M. 2017. Impacts of curatorial and research practices on the preservation of fossil hominid remains. Journal of Anthropological Sciences 95: 1–28. 27983518 10.4436/JASS.95002

[R35] Lelong O 2018. Fluid identities, shifting sands: Early Bronze Age burial at Cnip Headland, Isle of Lewis. Scottish Archaeological Internet Reports 75.

[R36] Lohof R 1994. Tradition and change: burial practices in the Late Neolithic and Bronze Age in the north-eastern Netherlands. Archaeological Dialogues 1: 98–118.

[R37] Mauduit A, Chenal F, Pélissier A, Barrand-Emam H. 2022. Les pratiques funéraires en Alsace du Néolithique à l’époque moderne. Lecture typo-chronologique des sépultures à inhumation. In: Blanchard P, Chimier J-P, Gaultier M. et Verjux C, Rencontre autour des typo-chronologies des tombes à inhumation. Actes de la 11e Rencontre du Gaaf du 3 au 5 juin 2019 à Tours. Publication du Gaaf n°11 (82e Supplément à la Revue Archéologique du Centre de la France): 37–50.

[R38] Mathieson I, 2015. Genome-wide patterns of selection in 230 ancient Eurasians. Nature 528: 499–503. 26595274 10.1038/nature16152 PMC4918750

[R39] Olalde I 2018. The Beaker phenomenon and the genomic transformation of northwest Europe. Nature 555: 190–196. 29466337 10.1038/nature25738 PMC5973796

[R40] Parker Pearson M 2005. Evidence for mummification in Bronze Age Britain. Antiquity 79: 529–546.

[R41] Parker Pearson M 2008. The powerful dead: archaeological relationships between the living and the dead. Cambridge Archaeological Journal 3: 203–229

[R42] Rathmann H, Stoyanov R, Posamentir R. 2022. Comparing individuals buried in flexed and extended position at the Greek colony of Chersonesos (Crimea) using cranial metric, dental metric, and dental nonmetric traits. International Journal of Osteoarchaeology 32: 49–63.

[R43] Rebay-Salisbury K, Stig Sørensen ML, Hughes J. 2010. Body parts and Bodies Whole: changing relations and meanings. Oxbow Book: Oxford.

[R44] Ringbauer H 2023. Accurate detection of identity-by-descent segments in human ancient DNA. Nature Genetics 10.1038/s41588-023-01582-w PMC10786714 38123640

[R45] Robb J, Elster ES, Isetti E, Knüsel CJ, Tafuri MA, Traverso A. 2015. Cleaning the dead: Neolithic ritual processing of human bone at Scaloria Cave, Italy. Antiquity 89: 36–54.

[R46] Rohland N, 2018. Extraction of highly degraded DNA from ancient bones, teeth, and sediments for high-throughput sequencing. Nature Protocols 13: 2447–2461. 30323185 10.1038/s41596-018-0050-5

[R47] Rohland N, 2015. Partial uracil-DNA-glycosylase treatment for screening of ancient DNA. Phylosophical Transactions of the Royal Society B Biological Sciences 370: 20130624 10.1098/rstb.2013.0624 PMC4275898 25487342

[R48] Salanova L, Brunet P, Cottiaux R, Hamon T, Langry-François F, Martineau R, Pollini A, Renard C & Sohn M. 2011. Du Néolithique récent à l’âge du Bronze dans le centre nord de la France : les étapes de l’évolution chrono-culturelle. In: Bostyn F, Martial E & Praud I (dir.), Le Néolithique du nord de la France dans son contexte européen, dans Revue archéologique de Picardie, n° spécial 28: 77–100.

[R49] Smits L & Van der Plicht J. 2009. Mesolithic and Neolithic human remains in the Netherlands: physical anthropological and stable isotope investigations. Journal of Archaeology of the Low Countries 1: 55–85.

[R50] Toussaint M 2003. Le “Champ mégalithique de Wéris”. Fouilles de 1979 à 2001. Volume 1. Contexte archéologique et géologique, Namur, Études et Documents, Archéologie 9.

[R51] Toussaint M 2013. L’archéologie en Wallonie. Le Néolithique. Carnet du Patrimoine 110. Namur: Institut du Patrimoine wallon.

[R52] Toussaint M, Collet H, Jadin I, Lavachery P, Pirson S, Woodbury M, Durieux J, Éloy J, Lambermont S. 2019. Recent discoveries of human skeletons in the flint mineshafts of Spiennes: casualties or burials. Anthropologica et Praehistorica 129: 245–262.

[R53] Triantaphyllou S 2016. Staging the manipultion of the derad in Pre- and Protopalatial Crete, Greece (3rd -early 2nd mill. BCE): from body wholes to fragmented body parts. Journal of Archaeological Science: Reports 10: 769–779.

[R54] Van der Velde H, Altena E, d’Hollosy M, Kootker L, Pijpelink A, Geerts R, Veldman A. 2022. Op zoek naar nieuw geluk. Onderzoek naar inhumaties uit de Romeinse tijd in Tongeren. Amersfoort: SYNTAR 10.

[R55] Veselka B 2023. Gallo-Roman cremation deposits from Pommeroeul, Belgium in La nécropole gallo-romaine sud de Pommerœul (province de Hainaut, Belgique) (ed. Cattelain L) 109–113. Viroinval: Cedarc.

[R56] Watermann AJ, Thomas JT. 2011. When the bough breaks: childhood mortality and burial practice in Late Neolithic Atlantic Europe. Oxford Journal of Archaeology 30:165–183.

[R57] Wojcieszak M, Van den Brande T, Ligovich G, Boudin M. 2020. Pretreatment protocols performed at the Royal Institute for Cultural Heritage (RICH) prior to AMS 14C measurements. Radiocarbon 62: e14–e24.

