# Supplementary material for: Assembling Ancestors: the manipulation of Neolithic and Gallo-Roman skeletal remains from Pommerœul, Belgium
Source: Antiquity. Author manuscript; Available in PMC 2024 Dec 19. (PMC11658144; doi:10.15184/aqy.2024.158)
Supplement: Appendix 1. Materials and Methods [file NIHMS2033442-supplement-Appendix_1__Materials_and_Methods.docx]

**Materials and Methods**

Grave 26 was discovered in the 1970s as part of the excavation of the Gallo-Roman settlement of Pommerœul, Belgium, in the course of using a soil-drill to investigate the deeper layers of the necropolis (pers. comm. M. Paumen, J. Wargnies, and A. Demory, the original excavators; Figure 1). The individual was buried in a flexed position on the right side, with the lower legs and feet scattered around the individual on about the same level (pers. comm. Paumen, Wargnies and Demory). At the back of the cranium, a bone pin, circular in cross-section, was retrieved, typologically dated to the 3^rd^ century AD (Riha 1990). The excavators suggested the remains to be female and to be accompanied by the remains of a nonadult. The ‘nonadult’ bones were later identified as badger remains, including one skull fragment, one first phalanx from an adult, and one humerus with unfused epiphyses from an immature individual. The badger phalanx was burnt.


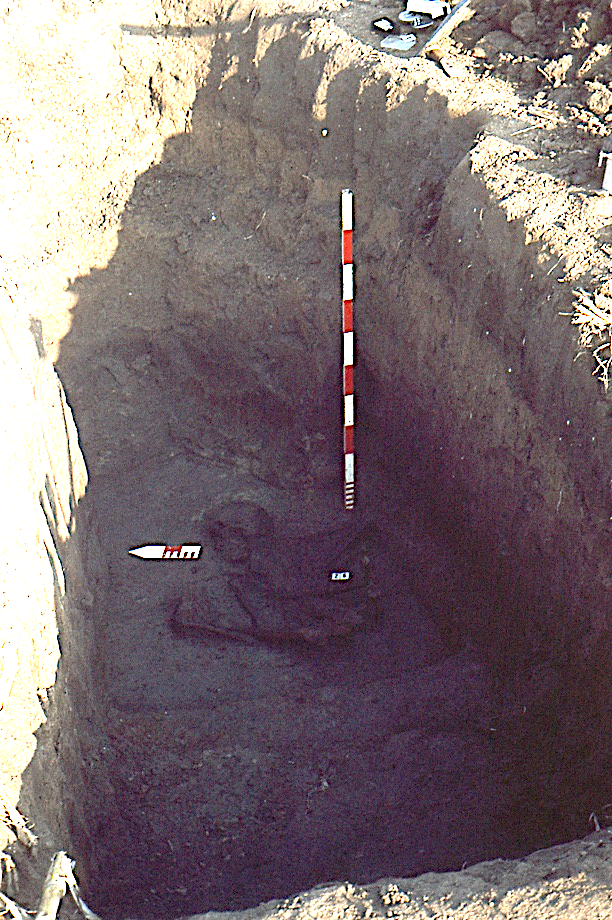


Figure 1. Photograph of grave 26 by the excavators.

An osteological sex determination was carried out using methods outlined in the Workshop of European Anthropologists (Ferembach *et al.* 1980), evaluating the Phenice traits (Phenice 1969), and measurements of the humerus, clavicles, and femur (McCormick *et al.* 1991; Stewart 1979; Steijn & Isçan 1999). Age-at-death was estimated via changes to the pubic symphysis (Brooks & Suchey 1990) and auricular surface (Buckberry & Chamberlain 2002) and evaluating the degree of cranial suture closure (Meindl & Lovejoy 1985). The bones were macroscopically assessed for traces of human modification, using the classification as described by Bello *et al*. (2016; and the references therein), thereby distinguishing slicing cut marks, scrape-marks, chop marks, percussion damage, and tooth marks.

To assess whether all the skeletal remains are contemporaneous, the humerus (left and right), femur (left and right), tibia (left and right), fibula (right) and five metatarsals, were sampled for radiocarbon dating, resulting in a total of 12 samples. Additionally, samples from the two different badger skeletal elements and the bone pin were taken. Radiocarbon dating was carried out at the Royal Institute for Cultural Heritage, Brussels (KIK-IRPA) and followed the KIK-IRPA protocol (Wojcieszak et al. 2020). All the measurements were obtained with the AMS type MICADAS, mini carbon dating system, at the KIK-IRPA (Lab-code: RICH; Boudin *et al.* 2015). Calibration of the radiocarbon ages (BP) into calendar years (BC/AD) was performed using the software OxCal 4.4 (Bronk Ramsey 2009) and the atmospheric calibration curve IntCal20 (Reimer *et al.* 2020). The consistency of the time series for the human bones was tested via a χ^2^-test (Ward & Wilson 1987) using the R_Combine tool in OxCal 4.4.

The radiocarbon dates were combined with aDNA analysis to not only evaluate if the bones belonged to contemporaneous individuals, but also to determine the Minimum Number of Individuals (MNI) that contributed to the interred individual and so assess possible kinship between the individuals. For this purpose, all major skeletal elements were sampled by cutting a wedge midshaft of each long bone and sampling the whole pars petrosa from the cranium.

In clean room conditions at Harvard Medical School, a sterile dentistry drill was used to obtain powder from 15 skeletal samples from Pommerœul. DNA was extracted from approximately 37.5 mg of powder from each sample (Dabney *et al.* 2013; Rohland *et al.* 2018; Korlević *et al.* 2015). The extracted DNA from the 15 samples was built into 16 double-stranded libraries, which were treated with the enzyme Uracil-DNA Glycosylase to remove characteristic error patterns associated with ancient DNA (Rohland *et al.* 2015). The libraries were enriched in solution for sequences overlapping about 1.2 million single nucleotide polymorphisms (SNPs; Fu *et al*. 2015) as well as the mitochondrial genome (Fu *et al.* 2013; Rohland *et al.* 2015; Mathieson *et al.* 2015; Rohland *et al.* 2022). The libraries were sequenced on the Illumina NextSeq500 instruments for 2 x 76 cycles or on Illumina HiSeqX10 instruments for 2 x 101 cycles, reading out the indices with 2 x 7 cycles in each case. Computational processing of the data and assessment of ancient DNA authenticity was performed as described in previous publications (Rohland *et al*. 2022). The results of this screening are in presented in Online Tables 1-3 (Appendix 2,4 and 5). The libraries were marked as failing quality control if they produced < 3000 SNPs covering the targeted positions. This left data for six individuals from Pommerœul that are published here. Three individuals had good quality data: I18605 with data from 616,997 targeted autosomal SNPs, I21570 with 159,871 SNPs, and I18068 with 84,775 SNPs. Data from three individuals with more marginal results are also reported: I18067 with 16,795 SNPs, I21568 with 4345 SNPs, and I21565 with 2981 SNPs. Finally, data from the two individuals from the Gallo-Roman cemetery of Tongeren (Belgium) were added, with genome-wide data at 751,084 SNPs (sample I21059) and 667,842 SNPs (sample I21058) was produced using the same methodology.

The genetic data was tested to determine whether the samples were consistent with being from the same person using the same algorithm as employed by the READ software (Monroy *et al.* 2018). The first measurement for this analysis is the rate *Q_ij_* at which two samples *i* and *j* mismatch each other at analyzed SNPs, at positions where they both have data from at least one sequence. The second measurement is the average rate *R* of mismatch of two randomly chosen sequences within the same individuals whose parent we assume are not closely related. Since two randomly chosen sequences from the same individual have a 50% chance of coming from the same parent, 2*R* is the mismatch rate expected for unrelated pairs of individuals and *R* is the mismatch rate expected for the same individual, and the degree of relatedness is quantified as *S_ij_* = 1-((*Q_ij_*-*R*)/*R*), a quantity that will range from 0 for unrelated to 1 for DNA from the same individual. A standard error can also be computed with a Block Jackknife. If *S_ij_* is confidently less than 1, the possibility that samples *i* and *j* can be excluded as being from the same person. For comparison between individuals without steppe ancestry, *R* = 0.120 is used, and for comparisons including the individual with steppe ancestry I19605, *R* = 0.131 is used.

A Principal Component Analysis (PCA) on 999 previously published present-day West-Eurasian individuals genotyped on the Affymetrix Human Origins SNP array was carried out using the *smartpca* module of the EIGENSOFT software (version 7.2.1.; Patterson *et al.* 2006)). Details of the modern individuals and the individual papers in which their data were published are reported in Online Table 2. The newly analysed ancient individuals were projected onto the modern genetic variation using the options lsqproject: YES and shrinkmode: YES.

To obtain 95% confidence intervals on the date separation Δt in generations between two individuals sharing a single segment of long IBD (quantified by genetic length “l” in units of Morgans), a likelihood-based framework was used. Specifically, the probability of observing that two individuals share an IBD of length given a date difference of Δt between the pair of individuals, Pr(IBD of length *l* | Δt), following the framework of Ringbauer *et al.* (2021) to obtain the expected number of IBD of length l. This framework computes the density of the expected number of IBD of length l (see Supplement Information, Equ. 7) as:

$$ƒ\left( l \right)dl\int_{0}^{\infty} dt b\left( l | t \right)\psi\left( t \right)dl$$

where $\psi\left( t \right)$ denotes the single locus coalescent probability, and $b\left( l | t \right)$ is the expected number of overlapping of blocks of length  *l*  caused by recombination *t* generations ago. The latter, $b\left( l | t \right)$, is a simple analytic expression, given explicitly by Ringbauer *et al.* (2021)

$\psi\left( t \right)$ is approximated by assuming that both samples are part of a population of size N_e_. If the two samples are separated by Δt generations, coalescence becomes possible at the generation of the earlier sample, and the single locus coalescence probability then becomes the single locus probability of a panmictic population:

$$\psi\left( t \right)=exp(-\frac{t}{Ne})\frac{1}{2Ne}$$

when time t is measured in generations before the earlier sample. Plugging that $\psi\left( t \right)$into the above integral makes it possible to calculate $ƒ\left( l \right)$. The probability of observing an IBD of length l, Pr(IBD of length l | Δt), can be approximated as proportional to this quantity $ƒ\left( l \right)$because, in the limit of small IBD length bins, the probability of observing an IBD in a length bin converges to a Bernoulli distribution with the chance of success equaling the expected value. Here, the constant of proportionality is ignored, as log-likelihood-based inference schemes remain invariant to such constant terms. These approximations allow to calculate Pr(IBD of length l | Δt) for various Δt, that is, the likelihood. To now obtain 95 percent confidence intervals for Δt, we use the standard 1.92 rule for log-likelihoods, estimating that the 95% confidence interval of a single parameter is approximately equal to the interval where the log-likelihood remains within 1.92 units of the maximum log-likelihood (Rossi 2018).

We applied this approach to estimate confidence intervals on the date difference between the Pommereoul sample (I18605) and the long IBD that it shares with the two Tongeren siblings. We treat the Tongeren full siblings as a single IBD sharing event, as it is the same IBD segment that is shared.

Setting the shared IBD length to the inferred length of 0.160 Morgan, we obtain a 95% confidence interval of separation of up to 28 generations apart (see Fig. SX). We note that we assumed a constant population size of N_e_=10,000. However, setting N_e_=1,000 or 100,000 gives the same confidence interval, likely because of the fact that on the timeframe relevant for sharing long IBD all but extremely small N_e_ produce effectively constant coalescent probabilities across generations.


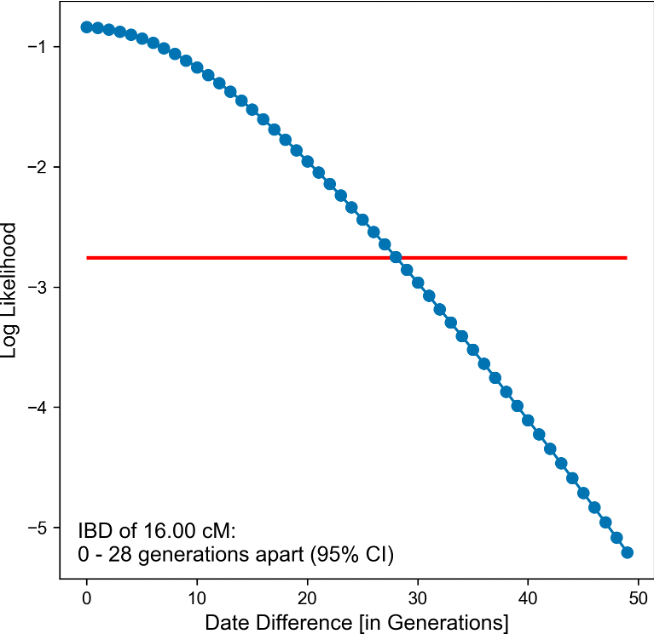


Figure 2. SX: Log-likelihood curve of date difference between Pommereoul and the two Tongeren full siblings.

**References**

Boudin, M., M. Van Strydonck, T. Van den Brande, H.A. Synal, L. Wacker. 2015. RICH – a new AMS facility at the Royal Institute for Cultural Heritage, Brussels, Belgium. *Nuclear Instruments Methods Phys Res, Sect B* 361: 120-123.

Brooks, S., B. Suchey. 1990. Skeletal age determination based on the os pubis: a comparison of the Ascádi-Nemeskéri and Suchey-Brooks methods. *Human Evolution* 5: 227-238.

Buckberry, J.L., A.T. Chamberlain. 2002. Age estimation from the auricular surface of the ilium: a revised method. *American Journal of Biological Anthropology* 119: 231-239.

Bronk Ramsey, C. 2009. Bayesian analysis of radiocarbon dates. *Radiocarbon* 51: 337-360.

McCormick, W.F., J.H. Stewart, F. Greene. 1991. Sexing of human clavicles using length and circumference measurements. *American Journal of Forensic Medicine and Pathology* 12: 175-181.

Dabney, J. *et al.* 2013. Complete mitochondrial genome sequence of a Middel Pleistocene cave bear reconstructed from ultrashort DNA fragments. *PNAS* 110: 15758-15763.

Ferembach, D. *et al*. 1980. Recommendations for age and sex diagnoses of skeletons. *Journal of Human Evolution* 9: 517-549.

Fu, Q. *et al.* 2013. DNA analysis of an early modern human from Tianyuan Cave, China. *PNAS* 110: 2223-2227.

Fu, Q. *et al*. 2015. An early modern human from Romania with a recent Neanderthal ancestor. *Nature* 524: 216-219.

Haak, W. *et al*. 2015. Massive migration from the steppe was a source for Indo-European languaes in Europe. *Nature* 522: 207-211.

Korlevi[ć](https://www.future-science.com/doi/full/10.2144/000114320), P. *et al*. 2015. microbial and human contamination in DNA extractions from ancient bones and teeth. *BioTechniques* 59: 87-83.

Mathieson, I. *et al.* 2015. Genome-wide patterns of selection in 230 ancient Eurasians. *Nature* 528: 499-503.

Meindl, S., O. Lovejoy. 1985. Ectocranial suture closure: a revised method for the determination of skeletal age at death based on the lateral-anterior sutures. *American Journal of Biological Anthropology* 68: 57-66.

Monroy Kuhn, J.M., M. Jakobsson, T. Günther. 2018. Estimating genetic kin relationships in prehistoric populations. *PLoS ONE* 13: e0195491.

Patterson, N., A.L. Price, D. Reich. 2006. Population structure and eigenanalysis. *PLoS genetics* 2: e190.

Phenice, T.W. 1969. A newly developed visual method of sexing the os pubis. *American Journal of Biological Anthropology* 30: 297-301.

Reimer, P.J. *et al.* 2020. The IntCal20 northern hemisphere radiocarbon age calibration curve (0-55 cal kBP). *Radiocarbon* 62: 725-757.

Riha, E. 1990. Der römische Schmuck aus August und Kaiseraugust. Forschungen in Augst 10.

Ringbauer, H., J. Novembre, M. Steinrücken. 2021. Parental relatedness through time revealed by runs of homozygosity in ancient DNA. *Nature Communications* 12: 1-11.

Rohland, N., E. Harney, S. Mallick, S. Nordenfelt, D. Reich. 2015. Partial uracil-DNA-glycosylase treatment for screening of ancient DNA. Phil. Trans. Royal Society B of Biological. Science 370: 20130624.

Rohland, N., I. Glocke, A. Aximu-Petri, M. Meyer. 2018. Extraction of highly degraded DNA from ancient bones, teeth, and sediments for high-throughout sequencing. *Nature protocols.* 13: 2447-2461.

Rohland, N. *et al*. 2022. Three assays for in-solution enrichment of ancient human DNA at more than a million SNPs. *Genome Res.* 32: 11-12.

Rossi, R.J. 2018. *Mathematical statistics: an introduction to likelihood-based inference*. John Wiley & Sons.

Steijn, M., M.T. Isçan. 1999. Osteometric variation in the humerus: sexual dimorphism in South Africans. *Forensic Science International* 106: 77-85.

Stewart, T.D. 1979. *Essential of forensic anthropology, especially as developed in the United States*. CC Thomas.

Ward, G.K., S.R. Wilson. 1987. Procedures for comparing and combining radiocarbon age determinations: a critique. *Archaeometry* 20: 19-31.

Wojcieszak, M., T. Van den Brande,G. Ligovich, M. Boudin. 2020. Pretreatment protocols performed at the Royal Institute for Cultural Heritage (RICH) prior to AMS ^14^C measurements. *Radiocarbon* 62:e14-e24.
