# Supplementary material for: Assembling Ancestors: the manipulation of Neolithic and Gallo-Roman skeletal remains from Pommerœul, Belgium
Source: Antiquity. Author manuscript; Available in PMC 2024 Dec 19. (PMC11658144; doi:10.15184/aqy.2024.158)
Supplement: Appendix 5. Overview skeletal elements_ [file NIHMS2033442-supplement-Appendix_5__Overview_skeletal_elements_.docx]

**Appendix 1.** Retrieved bones from grave 26 with estimated sex and age (adult vs. nonadult), and their position (anatomical vs. non-anatomical).

| Skeletal element | Sex | Age | Position |
| --- | --- | --- | --- |
| Cranium | F | Adult | Anatomical |
| Clavicle (left) | U | Adult | Anatomical |
| Clavicle (right) | U | Adult | Anatomical |
| Humerus (left) | U | Adult | Anatomical |
| Humerus (right) | U | Adult | Anatomical |
| Radius (left-gracile) | U | Adult | Anatomical |
| Radius (right) | U | Adult | Anatomical |
| Ulna (left) | U | Adult | Anatomical |
| Ulna (right) | U | Adult | Anatomical |
| Scapula (left) | U | Adult | Anatomical |
| 1^st^ Rib (left) | U | Adult | Anatomical |
| 1^st^ Rib (right) | U | Adult | Anatomical |
| 2^nd^ Rib (left) | U | Adult | Anatomical |
| 2^nd^ Rib (right) | U | Adult | Anatomical |
| 3^rd^ – 10^th^ Ribs (left; n = 9) | U | Adult | Anatomical |
| 3^rd^ – 10^th^ Ribs (right; n = 11) | U | Adult | Anatomical |
| Atlas | U | Adult | Anatomical |
| Axis | U | Adult | Anatomical |
| Cervical vertebrae  (n = 7) | U | Nonadult and adult | Anatomical |
| Thoracic vertebrae  (n = 5) | U | Nonadult and adult | Anatomical |
| Lumbar vertebrae  (n = 3) | U | Nonadult and adult | Anatomical |
| Os coxa (left) | M | Adult | Anatomical |
| Os coxa (right) | F | Adult | Anatomical |
| Femur (left) | M | Adult | Anatomical |
| Femur (right) | M | Adult | Anatomical |
| Patella (left) | U | Adult | Non-anatomical |
| Patella (right) | U | Adult | Non-anatomical |
| Tibia (left) | U | Adult | Non-anatomical |
| Tibia (right) | U | Adult | Non-anatomical |
| Fibula (right) | U | Adult | Non-anatomical |
| Capitate (left) | U | U | Non-anatomical |
| Capitate (right) | U | U | Non-anatomical |
| 2^nd^ Metacarpal (left; n = 2) | U | Adult | Anatomical |
| 3^rd^ Metacarpal (right) | U | Adult | Anatomical (but wrong side) |
| Proximal hand phalange (n = 15) | U | Adult | Non-anatomical |
| Intermediate hand phalange (n = 10) | M | Adult | Non-anatomical |
| Calcalneus (left) | U | Adult | Non-anatomical |
| Calcalneus (right) | U | Adult | Non-anatomical |
| Talus (left) | U | Adult | Non-anatomical |
| Talus (right) | U | Adult | Non-anatomical |
| Cuboid (right) | U | Adult | Non-anatomical |
| Medial cuneiform (left) | U | Adult | Non-anatomical |
| Intermediate cuneiform (left) | U | Adult | Non-anatomical |
| 1^st^ Metatarsal (left; n = 3) | U | Adult | Non-anatomical |
| 1^st^ Metatarsal (right; n = 5) | U | Adult | Non-anatomical |
| 2^nd^ Metatarsal (left; n = 2) | U | Adult | Non-anatomical |
| 2^nd^ Metatarsal (right) | U | Adult | Non-anatomical |
| 3^rd^ Metatarsal (right) | U | Adult | Non-anatomical |
| 4^th^ Metatarsal (left) | U | Adult | Non-anatomical |
| 4^th^ Metatarsal (right) | U | Adult | Non-anatomical |
| 5^th^ Metatarsal (left) | U | Adult | Non-anatomical |
| 5^th^ Metatarsal (right) | U | Adult | Non-anatomical |
| 1^st^ proximal foot phalanx (n = 2) | U | Adult | Non-anatomical |
| Proximal foot phalange (n = 6) | U | Adult | Non-anatomical |
| 1^st^ distal foot phalanx | U | Adult | Non-anatomical |
| 1^st^ proximal foot phalanx (n = 2) | U | Nonadult | Non-anatomical |
| Distal epiphyses femur (left and right) | U | Nonadult | Non-anatomical |

F = female, U = unobservable, M = male.
